# A case-control study to evaluate urinary tract complications in radical hysterectomy

**DOI:** 10.1186/1477-7819-3-12

**Published:** 2005-02-16

**Authors:** Nadereh Behtash, Fatemeh Ghaemmaghami, Haleh Ayatollahi, Hediyeh Khaledi, Parviz Hanjani

**Affiliations:** 1 Department of Gynaecology Oncology, Tehran University of Medical Sciences, Tehran, Iran; 2 Vali-e-Asr Reproductive Health Research Center, Tehran University of Medical Sciences, Tehran, Iran; 3 Department of Gynaecology and Obstetrics, Tehran University of Medical Sciences, Tehran, Iran; 4 Department of Gynaecology Oncology, Rosenfeld Cancer Center, Abington Memorial Hospital, Abington, PA, USA

## Abstract

**Background:**

This study has evaluated urinary tract injuries and dysfunction after Radical Hysterectomy (RH) performed in patients with cervical cancer and has compared the cystometric parameters and urinary complications occurring in these patients with those occurring in patients who had undergone Simple Hysterectomy (SH).

**Patients and methods:**

A prospective case-control study was conducted to evaluate urinary tract injuries (intra-operative and post-operative) and dysfunction in 50 patients undergoing RH for cervical cancer and to compare them with the same parameters in 50 patients who underwent SH for benign disease.

**Results:**

Mean age in the RH group was 46.3 years and in the SH group was 50.1 (p = 0.63). There were no bladder and urethral injuries in either group of patients. There was one intra-operative ureteral injury in the RH patients but none in those who underwent SH. (p < 0.05). In the two weeks after surgery, 15% of RH patients and 11% of SH patients had experienced a urinary tract infection urinary tract infection (p = 0.61). Two week after surgery 62% of RH patients had no urinary symptoms, compared to 84% in the SH group who did (p < 0.02). Urinary residual volume, first urinary sensation and maximal bladder capacity were higher in the RH group, but this was not statistically significant. The only case of a urinary fistula appeared in a patient who received 5000 cGy radiation therapy pre-operatively, but this spontaneously healed after 3 weeks of catheterization.

**Conclusions:**

Intra-operative and post-operative urinary tract complications are comparable in patients undergoing RH and SH and an expert gynaecological oncologist might be able to further decrease complications. However, radiation therapy before surgery may increase the risk of complications.

## Background

Although the incidence of lower urinary tract complications after RH has been reported with variable rates, up to one half of patients undergoing RH experience at least one lower urinary tract symptom that develops after surgery and at a variable period of time [1,2]. Several retrospective studies have examined lower urinary tract dysfunction and traumatic injuries in patients who have undergone RH [3,4].

In this study, we prospectively evaluated intra-operative urinary tract injuries in addition to post-operative urinary tract dysfunction and infection at 2, 6, and 14 weeks following surgery. We also compared these findings with those at the same times in patients who underwent SH for benign disease.

## Patients and methods

Between October 2000 and December 2002, 50 women who underwent RH and bilateral lymph node dissection (BLND) were considered eligible for inclusion in the study. All patients had squamous cell carcinoma of cervix (SCC Cx.) and were staged as being Stages I and II. The operations were performed by the same gynaecological surgeons, using the same standard technique (class III Piver & Rutledge). Pre-operative management was standardised for all patients. Preoperatively a detailed medical history, physical examination, routine laboratory tests, pelvic CT-scan (with intravenous and oral contrast), urine analysis, and urine culture were carried out.

The exclusion criteria were; a history of voiding dysfunction, previous pelvic surgery, brain or spinal cord diseases, diabetes mellitus, and contraindications to urodynamic studies. The latter included a history of vesicoureteral reflux, hydronephrosis, frequent or recent urinary tract infection or urethral stricture. Patients received one pre-operative and three post-operative doses of a second generation cephalosporin (Cephazolin).

The duration of surgery, amount of intra-operative haemorrhage and the occurrence of any organ injuries were recorded. A Foley's catheter was inserted at the time of surgery and was left in place for two weeks after surgery. The patient's urinary catheter was removed when their post-voiding residual volume was less than 75 ml.

Water cystometry, urinalysis and urine culture were performed at 2, 6, 14 weeks after operation. The test for water cystometry was performed with the subject lying in a supine position. A 12F double-lumen catheter was introduced transurethrally into the bladder to withdraw residual urine. The pressure-volume relationship of the bladder was determined by filling the bladder with isotonic saline at a rate of 30–50 ml/min. The cystometry fill phase ceased when the patient experienced an urge to void urine, the first indication being leakage through the urethra, or a bladder volume of 600 ml (which ever occurred first). The volume at the termination of the fill-phase was designated as the maximum bladder capacity (MBC). We also assessed the bladder volume of each patient at their first desire to void (V desire, ml). Post-void residual urine volume (RU) was determined by transurethral catheterization after voiding had ceased. The presence or absence of any urinary symptoms was determined by both questionnaire and direct interview with the patient.

Fifty patients with benign disease who had underwent SH, were evaluated at the same time periods in the same way for comparison with the RH group of patients. In the SH group of patients, the Foley's catheter was inserted for 24 hours after operation. Data were analysed by SPSS statistical software using the chi-square and Student's 't' test for data analysis.

## Results

During this study, 50 patients with early stage cervical cancer and who underwent RH for cervical cancer and 50 patients who had undergone SH for benign disease were evaluated. Two patients in the RH group and 3 from the SH group were lost during the study. The mean ages and their BMIs (Body Mass Index) in two groups of patients were not statistically different. However, parity in the RH group was higher (p < 0.05) (Table 1). In the RH group, the stages for the cervical cancer were 65.1%, 23.2% and 11.6% for I, II_A _and II_B _stages, respectively. Patients who had stage I_B2 _or higher stages of cervical cancer received 4500–5000 cGy of irradiation pre-operatively. None of these patients received adjuvant radiation during the interval between surgery and performance of urodynamic studies.

**Table 1:** Comparison of characteristics of Radical and Simple Hysterectomy groups of patients.

**Characteristics**	**RH group**	**SH group**	**p**
**Mean age**	50.10	46.35	0.63
**BMI**	24.25	26.05	0.66
**Parity**	6	4	0.00
**Blood loss (ml)**	576 ml	416 ml	0.04
**Mean operative time (min)**	183. min	112. min	0.00

RH-Radical hysterectomy; SH-simple hyterectomy

In the SH group, the most common pathological conditions requiring hysterectomy were as follows; dysfunctional uterine bleeding (47.83%), uterine myoma (21.7%), ovarian cyst (10.8%), chronic pelvic pain (4.3%), adenomyosis (4.3%), endometrial cancer (4.35%), CIN (4.35%) and molar pregnancy (2.17%).

The average blood loss and mean operative time for both groups are shown in table 1. There were no bladder and urethral injuries occurring during the primary operation in either of the two groups of patients. One patient (with stage Ib_1 _cervical cancer) in the RH group had an intra-operative ureteral injury (which happened at the time of "unroofing" the distal part), and the ureteral anastomosis was carried out at that time. The urinary catheter and ureteral stent were removed four weeks after operation.

Another patient (with stage Ib_2 _cervical cancer) had received chemo-irradiation (5000 cGy) pre-operatively. She had a spontaneous urine leakage from the vagina approximately 2 months after surgery. A retrograde cystography revealed a minute vesico-vaginal fistula. After 3 weeks of continuous bladder drainage, the fistula resolved spontaneously and she had no urinary leakage at her follow-up visits.

Post-operative positive urine culture and urinary symptoms (dysuria, frequency, nocturia and dribbling) are showed in table 2. Urinary symptoms occurred more commonly in patients who had undergone pre-operative radiotherapy, but this difference was not statistically significant (table 3). The abnormal findings as regards water cystometry are shown in table 4.

**Table 2:** Postoperative urinary symptoms in RH and SH group of patients.

	**RH group**	**SH group**	**p**
**Positive U/C 1^st ^visit**	15%	11%	0.06
**Positive U/C 2^nd ^visit**	31%	20%	0.00
**Positive U/C 3^rd ^visit**	11%	9%	NS
**Urinary symptoms 1^st ^visit**	31%	20%	0.00
**Urinary symptoms 2^nd ^visit**	40%	34%	0.07
**Urinary symptoms 3^rd ^visit**	30%	33%	NS

U/C-Urine Culture ; NS-Not significant; RH-Radical hysterectomy; SH-simple hyterectomy

**Table 3:** Comparison of urinary symptoms between patients undergoing RH but with or without pre-operative radiotherapy.

**Urinary symptoms (Postoperatively)**	**RH group**	**XRT + RH**	**p**
**2 weeks, 1^st ^visit**	39%	44%	0.76
**6 weeks, 2^nd ^visit**	36%	44%	0.64
**14 weeks, 3^rd ^visit**	26%	44%	0.27

XRT-History of pre-operative radiotherapy; RH-Radical hysterectomy

**Table 4:** Relative frequency of RV, MC, FS, SI and UTI in the patients.

		**Abnormal RV (%)**	**Abnormal FS (%)**	**Abnormal MC (%)**	**Abnormal SI (%)**	**UTI (%)**
First visit*	RH	4	66	68	22	31
	SH	0	67	69	17	20
Second visit**	RH	0	58	61	20	31
	SH	0	66	70	16	20
Third visit***	RH	0	49^†^	65	17	11
	SH	4	72^†^	65	18	9

*After discharging the drain

**Four weeks after discharging the drain

***Eight weeks after discharging the drain

†P-value = 0.02

## Discussion

Modern surgical techniques have resulted in a decrease in the incidence of lower urinary tract complications occurring as a result of radical hysterectomy. In particular, in recent times, various surgical strategies have been developed to avoid damaging the inferior segment of the cardinal ligament as well as the terminal bundle in the uterosacral and pubocervicovesical ligaments. This has made it possible for patients' lower urinary functions to return more rapidly to their pre-operative states [2,5]. However, transient post-operative urinary dysfunction involving urinary storage and evacuation function continues to be of concern [6].

In a study by Zaino and colleagues intra-operative complications were reported to occur as being 4.5% urinary tract and 8.7% other organs (nervous, haemorrhage, intestinal) [7]. Ralph *et al*, reported a 6.6% rate of intra-operative urinary tract injuries during radical surgery for cervical carcinoma [2]. In our study, we had no intestinal or bladder injuries occurring during radical hysterectomy. The only ureteral injury (2%) occurred during "unroofing" of the distal ureter and this was recognised and repaired immediately.

Zaino *et al*, [7] reported a 20% risk of a post-operative urinary tract fistula after radical surgery [7] and this contrasts with a 4.4% risk of fistula in their series reported by Ralph [2]. In our study, the only fistula occurred in a patient who had received 5000 cGy radiotherapy before radical surgery, and with continuous bladder drainage for 3 weeks there was spontaneous healing of the fistula. The incidence of urinary tract infection (UTI) in our series was 11% by 14 weeks after surgery and this was comparable to that reported by Cardosi [8] but less than the figure of 20% documented by Abrao [9]. Also, Chen reported a 14% urinary tract infection rate following radical surgery [10].

In our study, urinary tract dysfunctions that followed radical surgery were that 4% had an abnormal post-voiding residual volume at the first post-operative visit. The first voiding sensation at the third visit was 49% and stress urinary incontinence was 17%. However, maximal capacity remained abnormal in 65% of cases by 14 weeks after surgery. Ralph *et al *reported that 67% of patients had impairment or absence of bladder sensation after a RH [2]. In the study from Chen *et al*, 84% of patients had an increased first desire to void and maximal capacity in the post-operative period [10].

Urinary symptoms in our study occurred in 20% 2 weeks after operation and which were higher in patients with pre-operative radiotherapy (although not statistically significant). Urinary symptoms remained high at the third post-operative visit, although they declined in patients who had undergone surgery alone.

In our study, the patients mean age; BMI, parity, operative time, and blood loss were higher in those undergoing RH. The mean age of our patients was higher than patients in the study by Vervest *et al*, (mean was 45 years) [11]. Also in this study [11] the patients BMI of 23.2 was lower than that of the patients in our study. Cystometric parameters and intra-operative and post-operative complications showed little difference between patients having either RH or SH. The small number of patients in our study could have biased the results. However, in spite of the different gravidity and days of bladder drainage in the two groups of patients, the overall complication rate is relatively low in the RH patients. The data in this study requires confirmation from future multicentric studies with greater numbers of patients.

In recent years, several studies support the role of a gynaecological oncologist who is specifically trained in such aspects of care and who can obtain optimal cytoreductive surgery in patients with ovarian carcinoma [12,13]. Therefore, it would seem that an experienced and appropriately trained gynaecological oncologist might achieve a complication rate for patients undergoing radical hysterectomy comparable with that reported by "general gynaecological surgeons..

## Competing interests

The author(s) declare that they have no competing interests.

## Authors' Contributions

**NB **carried out the surgery and participated in drafting the manuscript.

**FG **carried out follow-ups.

**HA **participated in the design of the study and helped to draft the manuscript.

**HK **helped in follow-ups and performed the statistical analyses.

**PH **participated in the design of the study and helped to draft the manuscript.

All authors read and approved the final manuscript.
